# Practices and Attitudes of Swiss Stakeholders Regarding Investigator-Initiated Clinical Trial Funding Acquisition and Cost Management

**DOI:** 10.1001/jamanetworkopen.2021.11847

**Published:** 2021-06-02

**Authors:** Stuart McLennan, Alexandra Griessbach, Matthias Briel

**Affiliations:** 1 Department of Clinical Research, Basel Institute for Clinical Epidemiology and Biostatistics, University of Basel and University Hospital Basel, Basel, Switzerland; 2 Institute of History and Ethics in Medicine, Technical University of Munich, Munich, Germany; 3 Institute for Biomedical Ethics, University of Basel, Basel, Switzerland; 4 Department of Health Research Methods, Evidence, and Impact, McMaster University, Hamilton, Ontario, Canada

## Abstract

**Importance:**

Randomized clinical trials (RCTs) are an essential method of evaluating health care interventions and a cornerstone for evidence-based health care. However, RCTs have become increasingly complex and costly, which is particularly challenging for independent investigator-initiated clinical trials (IICTs). IICTs have an essential role in clinical research, and it is important that efforts are made to ensure IICTs are adequately funded and are conducted cost-effectively.

**Objective:**

To examine the practices and attitudes of Swiss stakeholders regarding IICT funding acquisition and cost management.

**Design, Setting, and Participants:**

For this qualitative study, interviews were conducted in Switzerland between February and August 2020. The purposive sample comprised 48 stakeholders from 4 different groups: primary investigators (n = 27), funders and sponsors (n = 9), clinical trial support organizations (n = 6), and ethics committee members (n = 6).

**Main Outcomes and Measures:**

Practices and attitudes of stakeholders regarding IICT funding acquisition and cost management were assessed using individual semistructured qualitative interviews. Interviews were analyzed using conventional content analysis.

**Results:**

After interviews with 48 IICT stakeholders (75% male presenting), these participants identified a systemic problem of IICTs being underfunded, which can lead to compromises being made regarding the quality and conduct of IICTs. Participants identified 2 overarching and interconnected groups of reasons why IICTs in Switzerland are regularly underfunded. First, it was reported that IICT budget estimations are often inaccurate because of poor planning and preparation, unforeseeable events, investigators intentionally underestimating budgets, and limited budget assessment and oversight. Second, with the exception of a specific IICT funding program by the Swiss National Science Foundation, it was reported that limited funding sources and unrealistic expectation of funders led to underlying challenges in getting IICTs fully funded. A number of measures that could help reduce the underfunding of IICTs were identified, including improving the support of investigators and IICTs, strengthening networking and guidance, harmonizing and simplifying bureaucracy, and increasing public funding of IICTs.

**Conclusions and Relevance:**

This study highlights the inadequate expertise of Swiss stakeholders to correctly, systematically, and reproducibly calculate RCT budgets and the need for transparency on trial costs as well as training in budgeting practices. Limited financial resources for academic clinical research and issues regarding the professional planning and conduct of IICTs are persistent issues that many other countries also face.

## Key Points

**Question:**

What are the practices and attitudes of Swiss stakeholders regarding funding acquisition and cost management in investigator-initiated clinical trials (IICTs)?

**Findings:**

In this qualitative study, including interviews of 48 IICT stakeholders, respondents reported that they lacked experience and expertise in the budgeting of IICTs. A lack of responsibility for the quality assurance of this task was also identified.

**Meaning:**

These findings suggest that there is a need for empirical and transparent data on IICT costs, including budget monitoring and calculation tools, which may help avoid further conflict among stakeholders and promote adequate funding for IICTs.

## Teaser

This qualitative study examines practices and attitudes regarding funding acquisition and cost management of investigator-initiated clinical trials among primary investigators, funders and sponsors, clinical trial support organizations, and ethics committee members in Switzerland.

## Introduction

Randomized clinical trials (RCTs) are an essential method of evaluating health care interventions and a cornerstone for evidence-based health care.^1,2,3^ Conducting high-quality RCTs, however, is a challenging and resource-demanding enterprise typically associated with high costs.^4,5,6,7,8^ During the last decades, various initiatives and regulations to improve participant protection and research quality have also increased the administrative burden and overall costs of RCTs.^1,9,10,11,12,13,14,15,16,17^ Furthermore, the implementation of medical advances, although highly desirable, has limited the incremental impact of new therapies. This has been associated with smaller beneficial effects of interventions and thus requiring larger RCT sample sizes.^18,19^ These developments have resulted in RCTs becoming increasingly complex and costly, aggravating existing practical challenges.

Given the limited resources available for academic clinical research, these cost pressures are particularly challenging for independent investigator-initiated clinical trials (IICTs)^20^ and have contributed to a decrease in the number of IICTs being conducted.^2,21,22^ Previous research examining over 1000 RCT protocols in Switzerland, Germany, and Canada also found that a substantial proportion of IICTs are prematurely discontinued due to recruitment and organizational problems, whereas industry-initiated RCTs were much less frequently discontinued; suggesting that adequate funding and professional planning and conduct of RCTs are crucial.^23^ Nevertheless, the medical literature lacks detailed empirical data on costs and resource use of IICTs, or an understanding of the practices and attitudes of key stakeholders regarding IICT funding and cost management.^7,8,24^

In Switzerland, the need to better practically support IICTs has been increasingly recognized; there has been a network of clinical trial units to support the high-quality conduct of academic clinical studies since 2007,^25^ and the Swiss National Science Foundation has had a yearly program for IICTs since 2016.^26^ However, a 2017 qualitative study reported that Swiss stakeholders still believe that most cases of trial discontinuation could be avoided through better planning and monitoring of participant recruitment and budget during the trial.^27^

IICTs have an essential role in clinical research, typically focusing on more patient-centered research questions taken from clinical care that have limited financial interest to industry.^20,28,29,30^ It is therefore important that efforts are made to ensure IICTs are adequately funded and are conducted cost-effectively. However, there is a need to better understand current practices and attitudes of investigators and other stakeholders. This study therefore sought to examine the practices and attitudes of Swiss stakeholders regarding IICT funding acquisition and cost management (budget estimation, budget assessment, and cost monitoring).

## Methods

Study design and data collection did not require approval of an ethical committee in Switzerland, per Article 1 and Article 2 of the Federal Act on Research Involving Human Beings. The methods of the study are presented in accordance with the Consolidated Criteria for Reporting Qualitative Research (COREQ) reporting guideline.^31^ See the eMethods in the Supplement for additional details on methods used in the study.

### Research Team and Reflexivity

Interviews were primarily conducted by S.M., a male postdoctoral researcher in biomedical ethics. One interview was conducted by M.B., a male physician and senior scientist in clinical epidemiology. Both interviewers have longstanding experience with qualitative research.^27,32,33,34,35,36,37,38,39,40,41,42,43,44,45^ The interviewers had already had contact with many of the stakeholders from previous research studies. Otherwise, no relationship was established between the interviewers and the other participants prior to the study, and participants received limited information about interviewers. There was no hierarchical relationship between the interviewers and the study participants.

### Study Design

Stakeholders were primarily selected through purposive sampling to ensure that participants involved in the IICTs were from different backgrounds.^46^ Additional participants were identified using snowball sampling.^47^ Participants were contacted by email and suitable dates for an interview were found with those willing to participate. Forty-eight stakeholders agreed to participate in the study and were recruited from 4 different groups: primary investigators (PIs), funders and sponsors, clinical trial support organizations, and ethics committee members. Interviews were held between February and August 2020. One participant provided their response in writing via email and one interview was conducted in person, the remaining interviews were conducted via a telephone or video call. All interviews were conducted in English. A researcher-developed, semistructured interview guide was developed for each group to guide the discussion (eAppendix in Supplement). Based on the first 2 interviews that did not show any problems, it was decided that no further piloting or adaptation of the interview guides was necessary. Interviews were audio recorded and lasted a median (range) time of 29 (12-62) minutes. After 48 interviews, it was concluded that saturation was reached in the attitudes expressed by the participants.^48^ Transcriptions of the interviews were returned to all participants with an invitation for them to review the transcription and send any corrections or clarifications; 6 responses were received with minor corrections to syntax.

### Data Analysis

Using the interview transcriptions, S.M. performed conventional content analysis with the assistance of the qualitative software MAXQDA version 11 (VERBI Software) from October to December 2020.^49^ Initial themes identified as common across participants as well as those unique to individuals were labeled using a process of open coding. Findings are presented as higher- and lower-level categories in a coding frame. The other investigators (A.G. and M.B.) reviewed the initial analysis to clarify and refine codes, and conversations among the investigators continued until consensus was achieved.

## Results

Among the 48 stakeholders who participated in the study, 27 were PIs, 9 were funders and sponsors, 6 were clinical trial support organizations, and 6 were ethics committee members; overall, 75% (36/48) of stakeholders were male presenting.

### The Problem

Participants identified a systemic problem of IICTs being substantially underfunded in Switzerland (see Table 1; eTable 1 in Supplement). Although participants noted a small number of exceptions, it was reported that most IICTs are unable to be completed as planned or within budget. The actual costs of IICTs were reported to be nearly always higher than what had been estimated, and that funding limitations often lead to compromises being made regarding the quality and conduct of the IICT. Commonly mentioned sacrifices included reducing the sample size, adapting the end point, shortening the length of follow-up, and aspects of project management such as quality and budget monitoring. Funding limitations were also reported to lead to a substantial amount of trial costs not being funded or reimbursed; it was regularly estimated that these so-called hidden costs represented approximately 20% of the trials total costs.

**Table 1.** The Problem

Theme	Subtheme
Underfunded IICTs	
Compromises made because of funding limitations	Reducing sample size
	Adapting end point
	Length of follow-ups
	Project management
Hidden costs	Significant amount of trial costs not reimbursed
	Unfunded work often not recorded

Abbreviation: IICT, investigator-initiated clinical trials.

### The Reasons

Participants identified 2 overarching and interconnected groups of reasons why IICTs in Switzerland are regularly underfunded. These reasons are explained here in addition to Table 2 and eTable 2 in the Supplement.

**Table 2.** The Reasons

Theme	Subtheme
Inaccurate budget estimates	
Poor planning and preparation	Lack of professional support with cost management
	Unsystematic and limited evidence-base
Unforeseeable events	NA
Intentional underestimating budgets	NA
Limited assessment and oversight of budget	Very superficial assessment
	Lack of explicit criteria
	Budget accuracy responsibility of the PI
Funding limitations	
Limited funding sources	SNSF’s IICT program main option for full funding
	Foundations only able to fund pilot studies or provide partial support
	IICTs often started without having secured sufficient funds
Unrealistic expectation of funders	Gross underestimation of costs
	Funders cutting budgets

Abbreviations: IICT, investigator-initiated clinical trials; NA, not applicable; PI, primary investigators; SNSF, Swiss National Science Foundation.

#### Inaccurate Budget Estimates

The majority of participants reported that IICT budget estimations are often inaccurate; in that they are overly optimistic and underestimating the actual work involved. Participants identified a number of factors that contributed to inaccurate budget estimates.

##### Poor planning and preparation

Participants reported that IICTs with inaccurate budgets were often poorly planned and prepared, particularly with regard to planning and monitoring patient recruitment. Poor planning and preparation manifested itself in 2 key forms:

- *Insufficient professional support*: It was reported that it is typically IICTs that do not receive external professional support to calculate the budget and to monitor costs that ended up being underfunded. These PIs typically rely on the assistance of internal administrators or graduate students, the advice of experienced colleagues, and the templates provided by funders. However, these PIs acknowledged that they often forget to plan for certain resources in the budget and do not have a good overview of their project’s costs.
- *Unsystematic and limited evidence base*: Large variation was reported regarding how systematic and evidence-based IICT planning and preparation was conducted. Although the majority of participants agreed that feasibility or pilot work was important, it was reported that it was often done informally or not at all. Furthermore, it was reported that many PIs’ and trial support organizations’ efforts to base their budget estimates on data was usually not done in a formalized or systematic way; and typically only based on internal data from their own department or the experience of colleagues. Two key reasons for not considering empirical cost data from other similar clinical trials were identified: (1) difficulties finding or accessing cost data and (2) the view that the data would not be comparable to their own setting.

##### Unforeseeable events

It was also noted by participants that given the duration and complexity of IICTs, unexpected events such as staff sickness, pandemics, and drug production problems can inevitability occur, causing delays to the trial. Because funders do not permit unforeseen costs to be budgeted for IICTs, these delays can lead to substantial budget overruns.

##### Intentionally underestimating budgets 

There were also reports of many PIs intentionally underestimating IICT budgets to increase their perceived chances of receiving funding. Rather than calculating budgets based on how much they actually need for the study, it was reported that PIs often calculate based on how much they think they can get from a funder. A sponsor of clinical trials reported that they no longer allow PIs to calculate their own budgets for this reason.

##### Limited budget assessment and oversight 

It was reported that funders’ and ethics committee members’ consideration of IICT applications focus primarily on scientific content, and that the assessment of budgets currently has a very superficial role. Although budgets are checked if they are realistic and sufficient for the proposed project, explicit criteria are not currently used to make this assessment; it is usually a judgement call based on previous personal experience. This was seen to be problematic by some funders given their lack of training in budgets. Whether a budget for an IICT is accurate and sufficient, however, was ultimately seen by the majority of funders and ethics committee members to be the responsibility of the PI. Cost monitoring oversight was also reported to be limited. Annual financial reports are usually requested by funders, but no systematic feedback is given on planned and actual costs.

#### Funding Limitations

Participants also reported 2 key underlying challenges in getting IICTs fully funded in Switzerland. These included:

##### Limited funding sources 

Participants reported limited options for obtaining funding for IICTs in Switzerland. This situation often requires PIs of Swiss IICTs to obtain funding from multiple sources. Although some PIs said they would not start recruiting patients before the trial is fully funded, it was acknowledged by many PIs that they would begin recruiting without having secured sufficient funds, with the need to obtain further funding to be able to finish the trial.

##### Unrealistic expectation of funders 

PIs and clinical trial support participants reported that it was extremely difficult to get realistic IICT budgets accepted due to Swiss funders grossly underestimating the costs of trials because of a lack of awareness or understanding. The practice of funders cutting realistic budgets was widely reported to have negative consequences, contributing to underfunded trials, compromising trial quality, and encouraging PIs to submit intentionally inaccurate budgets. Although there now exists a program aiming to fund actual costs of IICTs and that sets no limits on the amount of funding that can be awarded, it was reported that PIs perceptions of a cap continue to influence how much funding they request. This situation was reported to also undermine any argument to ask for more public money for the program.

### The Solutions

Participants identified a number of measures that could help reduce the underfunding of IICTs in Switzerland. (See Table 3 and eTable 3 in the Supplement).

**Table 3.** The Solutions

Theme	Subtheme
Improving planning and quality	
Support of clinical researchers	Ensuring clinical researchers have sufficient time for their IICTs
	Education and training
Clinical trial support	Improving clinical trial support organizations
	Developing support tools
Networking and guidance	Sharing knowledge and experiences
	Sharing cost data
	Information sources
Cost savings	
Not possible to save costs	NA
Possible cost savings	Harmonizing and simplifying bureaucracy
	Simplified and remote techniques for quality monitoring
Increasing funding	
Making sufficient funding available	Increasing public funding of IICTs
	More flexibility with use of funding

Abbreviations: IICT, investigator-initiated clinical trials; NA, not applicable.

#### Improving Planning and Quality

##### Support of clinical researchers

A number of participants expressed the need to better support PIs, particularly young clinical researchers, to do their clinical work and training together with an academic career and ensure that they have sufficient time for their IICTs. Participants also identified the general need for more education and training of clinical researchers to improve the quality of clinical trial protocols and grants. Funders in particular expressed the notion that many IICT protocols currently lack sufficient methodological quality and feasibility, and that as long as these problems persist, only few IICTs are actually worth funding.

##### Clinical trial support

Participants also felt that IICTs needed to be better supported:

- *Improving clinical trial support organizations:* PIs reported substantial variations in satisfaction with the services provided by the different clinical trial support organizations. Although PIs viewed the support of some local support organizations as essential, they viewed others as not helpful at all given that their budget estimates were not accurate, they did not pick up on critical issues in cost monitoring, and their services were seen as too expensive. This has led some PIs to no longer seek assistance from their local support organization. Participants identified the need for improving and harmonizing support organization services and increasing their funding.
- *Developing support tools:* Participants reported that formal budget calculation or cost monitoring tools are rarely used by PIs or clinical trial support organizations. However, internally developed spreadsheets—of varying degrees of sophistication—are widely used. Although some PIs were satisfied with the current approach, many participants identified this as an area that could be improved and felt that formal tools would be very helpful for IICT PIs to reduce mistakes and improve accuracy.

##### Networking and guidance

Participants expressed a strong desire for better networking and guidance to improve the cost-effectiveness of IICTs. Participants felt that there are too many PIs doing their own thing, and that there is a need for PIs to come together and share knowledge and experience with each other. Many PIs also expressed a strong wish to have access to empirical cost data to get a more accurate picture of IICT costs. Although a funder reported that they were unable to share such data because of data protection regulations, they noted the possibility of PIs networking among themselves. Participants also expressed support for more information sources for PIs, including a comprehensive overview of possible funding sources of IICTs, and explicit criteria and suggestions of plausible ranges for requested funds for certain tasks to help PIs develop realistic budgets.

#### Cost Savings

Although participants were generally of the view that further cost savings were not possible given that IICTs are already being done so cheaply, some identified 2 areas that they thought could be done more efficiently. First, administration and bureaucracy around getting approval from Swiss Medic and ethics committees were seen as a key cost driver because of the delays caused to trials. Although PIs accepted that these steps were necessary and important, they expressed a strong desire for bureaucracy to be harmonized and simplified to reduce time and money. Second, participants felt that using more simplified and remote techniques for quality monitoring was also a possible way that costs could be saved in IICTs.

#### Increasing Funding

Although improving the planning and cost efficiency of IICTs was seen as important, it was felt by some that such measures would only be helpful if sufficient funding is actually available. Ultimately, many of the participants were of the view that what was required to prevent unfunded IICTs was the increase of public funding. Desire was also expressed for more flexibility regarding the use of the funding, including less formal criteria or flexibility regarding trial duration and the activation date of a trial.

## Discussion

To our knowledge, this is the first qualitative study to examine the practices and attitudes of key stakeholders regarding IICT funding acquisition and cost management. Participants identified a systemic problem of IICTs being substantially underfunded in Switzerland, which often lead to compromises being made in trial quality and conduct. This situation was reported to be largely caused by interconnected issues around limited financial resources and the professional planning, support, and conduct of IICTs. Our results reflect persistent issues that are not all unique to IICTs and may also be found in other types of clinical studies (eg, poor planning or problems with unforeseeable events) and other countries.

The chronic underfunding of IICTs is ethically problematic. It can undermine the scientific quality of the research, risk trial discontinuation, waste limited public resources, waste patients’ contributions, and put patients at unnecessary risk.^50,51,52^

This study highlights the inadequate budgeting expertise, the lack of responsibility in ensuring appropriate budget planning, and the need for better coordination between relevant stakeholders. In particular, there is a need for more transparency regarding the actual costs of IICTs, which will help inform evaluators whether IICT funding is sufficient, and whether IICTs should be allowed to be conducted if under a certain funding threshold.

Given the duration and complexity of IICTs, unpredictable events can inevitability occur, causing delays in trial duration.^53^ Stakeholders should discuss how best to address these issues and minimize the damages. This may include establishing emergency funds, granting extensions or budgeting for emergency provisions in grant applications. Furthermore, greater support and collaboration around planning and preparation of IICTS is essential. Consideration should also be given to modern trial designs, which show increased output efficiency and reduction in trial costs due to shared trial infrastructure and patient screening.^54,55^

### Strengths and Limitations

This is a qualitative study that did not collect statistically representative data. However, we included a range of experts who have direct experience with IICT funding acquisition and cost management in Switzerland, which makes it likely that this study has captured key aspects of a multi-sided conflict. A bias might exist toward the reporting of socially desirable attitudes.^56^ Given our results that are rather critical of current practice, we believe that such a bias is limited. The study was only carried out in Switzerland, and there may be some country-specific differences that might limit the generalizability. Nevertheless, many of the key issues are associated with aspects that are common in other countries (eg, limited financial resources available for academic clinical research, and issues regarding the professional planning and conduct of IICTs), these findings are likely to be of global interest. The strengths of this study include that it is, to our knowledge, one of the first to investigate stakeholder attitudes and current budgeting practices in IICTs. We ensured that all opinions were represented and included a broad range of relevant parties.

## Conclusions

This study found inadequate expertise concerning the calculation and assessment of IICT budgets in Swiss stakeholders and provided interconnected reasons that may explain the present situation. These findings suggest that there is a need for more transparency on trial costs as well as training in budgeting practices.

## Notes

Supplement. **eMethods.** Extended Methods eReferences **eAppendix.** Interview Guides **eTable 1.** The Problem **eTable 2.** The Reasons **eTable 3.** The Solutions
