# Supplementary material for: Practices and Attitudes of Swiss Stakeholders Regarding Investigator-Initiated Clinical Trial Funding Acquisition and Cost Management
Source: JAMA Netw Open. 2021 Jun 2;4(6):e2111847. doi: 10.1001/jamanetworkopen.2021.11847 (PMC8173375; doi:10.1001/jamanetworkopen.2021.11847)
Supplement: Supplement. — eMethods. Extended Methods eReferences eAppendix. Interview Guides eTable 1. The Problem eTable 2. The Reasons eTable 3. The Solutions [file jamanetwopen-e2111847-s001.pdf]

Supplemental Online Content

McLennan S, Griessbach A, Briel M; Making Randomized Trials Affordable (MARTA) Group. Practices and attitudes of Swiss stakeholders regarding investigator-initiated clinical trial funding acquisition and cost management. *JAMA Netw Open.* 2021;4(5):e2111847. doi:10.1001/jamanetworkopen.2021.11847

eMethods. Extended Methods

eReferences

eAppendix. Interview Guides

eTable 1. The Problem

eTable 2. The Reasons

eTable 3. The Solutions

This supplemental material has been provided by the authors to give readers additional information about their work.

© 2021 McLennan S et al. *JAMA Network Open.*

eMethods: Extended Methods

The methods of the study are presented in accordance with the "Consolidated criteria for reporting qualitative research" (COREQ) [1].

Research team and reflexivity

Personal characteristics: Interviews were primarily conducted by S.M., a male Post Doc in biomedical ethics. One interview was conducted by M.B., a male physician and senior scientist in clinical epidemiology. Both interviewers have longstanding experience with qualitative research [2-16].

Relationship with participants: The interviewers had already had contact with a number of the stakeholders from previous research studies. Otherwise, no relationship was established between the interviewers and the other participants prior to the study and participants received limited information about interviewers. There was no hierarchical relationship between the interviewers and the study participants.

Study design

Theoretical framework: The theoretical framework employed in this study was conventional content analysis [17].

Participant selection: Stakeholders were primarily selected through purposive sampling [18]; to ensure that participants involved in the IICTs were from different backgrounds. Additional participants were identified using snowball sampling [19]. Participants were contacted by email and suitable dates for an interview were found with those willing to participate. Forty-eight stakeholders agreed to participate in the study and were recruited from four different groups: primary investigators (PIs) of IICTs (n=27), funders and sponsors of IICTs (n=9), clinical trial support organisations (n=6), and ethics committee members (n=6). Four people refused to participate because they did not think they were the suitable person or because of workload issues.

Setting: Interviews were held between February and August 2020. One participant provided their response in writing via email, the remaining interviews were conducted via a telephone or video call. All interviews were conducted in English. Only the participant and the researcher were present during the interview. Overall, 75% (36/48) of stakeholders were male presenting, and 25% (12/48) were female presenting.

Data collection: A researcher-developed semi-structured interview guide was developed for each group to guide the discussion (see Supplementary Table 1). Based on the first two interviews that did not show any problems, it was decided that no further piloting or adaptation of the interview guides was necessary. No repeat interviews were carried out. Interviews were audio recorded, no field notes were taken. Interviews lasted an average of 29 minutes (range 12 minutes – 62 minutes). After 48 interviews the question about data saturation arose and it was concluded that saturation was reached in the content and attitudes expressed by the participants [20]. Transcriptions of the interviews were returned to all participants with an invitation for them to review the transcription and send any corrections or clarifications; six responses were received with minor corrections to syntax.

Analysis and findings

Using the interview transcriptions in their original language, S.M. performed conventional content analysis with the assistance of the qualitative software MAXQDA v11. Analysis commenced while interviews were ongoing. Initial themes identified common across participants as well as those unique to individuals were labelled using a process of open coding. Findings are presented as higher- and lower-level categories in a coding frame. The other investigators [A.G., M.B.] reviewed the initial analysis to clarify and refine codes, and conversations among the investigators continued until coding differences were resolved and consensus was achieved.

eReferences

1. Tong A, Sainsbury P, Craig J. Consolidated criteria for reporting qualitative research (COREQ): a 32-item checklist for interviews and focus groups. Int J Qual Health C 2007; 19: 349-57.
2. Briel M, Elger B, von Elm E, Satalkar P. Insufficient recruitment and premature discontinuation of clinical trials in Switzerland: qualitative study with trialists and other stakeholders. Swiss Med Wkly 2017;147:w14556.
3. Gloy V, McLennan S, Rinderknecht M, Ley B, Meier B, Driessen S, Gervasoni P, Hirschel B, Benkert P, Gilles I, von Elm E, Briel M. Uncertainties about the need for ethics approval in Switzerland: a mixed-methods study. Swiss Med Wkly. 2020 Aug 12;150:w20318. doi: 10.4414/smw.2020.20318. PMID: 32799307.

© 2021 McLennan S et al. *JAMA Network Open.*

4. Briel M, Speich B, von Elm E, Gloy V. Comparison of randomized controlled trials discontinued or revised for poor recruitment and completed trials with the same research question: a matched qualitative study. Trials. 2019 Dec 30;20(1):800. doi: 10.1186/s13063-019-3957-4. PMID: 31888725; PMCID: PMC6937940.
5. von Niederhäusern B, Schandelmaier S, Mi Bonde M, Brunner N, Hemkens LG, Rutquist M, Bhatnagar N, Guyatt GH, Pauli-Magnus C, Briel M. Towards the development of a comprehensive framework: Qualitative systematic survey of definitions of clinical research quality. PLoS One. 2017 Jul 17;12(7):e0180635. doi: 10.1371/journal.pone.0180635. PMID: 28715491; PMCID: PMC5513422.
6. Kasenda B, Liu J, Jiang Y, Gajewski B, Wu C, von Elm E, Schandelmaier S, Moffa G, Trelle S, Schmitt AM, Herbrand AK, Gloy V, Speich B, Hopewell S, Hemkens LG, Sluka C, McGill K, Meade M, Cook D, Lamontagne F, Tréluyer JM, Haidich AB, Ioannidis JPA, Treweek S, Briel M. Prediction of RECRUITment In randomized clinical Trials (RECRUIT-IT)-rationale and design for an international collaborative study. Trials. 2020 Aug 21;21(1):731. doi: 10.1186/s13063-020-04666-8. PMID: 32825846; PMCID: PMC7441612.
7. McLennan S. Rejected Online Feedback from a Swiss Physician Rating Website between 2008 to 2017: Analysis of 2352 Ratings. Journal of Medical Internet Research. 2020;22(8):e18374.
8. McLennan S. The Content and Nature of Narrative Comments on Swiss Physician Rating Websites: Analysis of 849 Comments. Journal of Medical Internet Research. 2019;21(9):e14336.
9. McLennan S. The ethical oversight of learning health care activities in Switzerland: a qualitative study. International Journal for Quality in Health Care. 2019; 31;31(8):G81-G86.
10. McLennan S, Moore, J. New Zealand District Health Boards' Open Disclosure Policies: A Qualitative Review. Journal of Bioethical Inquiry. 2019; 16(1): 35-44.
11. McLennan S, Strech D, Kahrass H. Why are so few patients rating their physicians on German physician rating websites? A qualitative study. BMC Health Services Research. 2018; 18:670.
12. McLennan S, Kahrass H, Wieschowski S, Strech D, Langhof H. The spectrum of ethical issues in a Learning Health Care System: a systematic qualitative review. International Journal for Quality in Health Care. 2018; 30(3): 161–168.
13. McLennan S, Schwappach D, Harder Y, Staender S, Elger B. Patient Safety Issues in Office-based Surgery and Anaesthesia in Switzerland: A Qualitative Study. Zeitschrift für Evidenz, Fortbildung und Qualität im Gesundheitswesen. 2017; 125: 23-29.
14. Pless A*, McLennan S*, Nicca D, Shaw D, Elger B. Reasons why Nurses Decline Influenza Vaccination: a Qualitative Study. BMC Nursing. 2017:16: 20.
15. Pless A, Shaw D, McLennan S*, Elger E.* Nurses' Attitudes towards Enforced Measures to Increase Influenza Vaccination: a Qualitative Study. Influenza and Other Respiratory Viruses. 2017;11(3):247-253.
16. McLennan S, Diebold M, Rich LE, Elger B. Nurses' Perspectives Regarding the Disclosure of Errors to Patients: A Qualitative Study. International Journal of Nursing Studies. 2016;54:16-22.
17. Hsieh HF, Shannon SE. Three approaches to qualitative content analysis. Qualitative Health Res. 2005;15(9):1277–88.
18. Palinkas LA, Horwitz SM, Green CA, Wisdom JP, Duan N, Hoagwood K. Purposeful Sampling for Qualitative Data Collection and Analysis in Mixed Method Implementation Research. Adm Policy Ment Health. 2015; 42(5): 533-44.
19. Marshall MN. Sampling for qualitative research. Fam Pract. 1996;13(6):522-5.
20. Fusch PI, Ness LR. Are We There Yet? Data Saturation in Qualitative Research. The Qualitative Report. 2015; 20(9): 1408-1416.

© 2021 McLennan S et al. *JAMA Network Open.*

eAppendix: Interview Guides

Primary investigators interview guide

1. To start, could you tell me a bit about the types and how many clinical trials you have conducted?
    a. Were these single or multi-center clinical trials?

….

4. Did you consider conducting a pilot clinical trial or other feasibility work?
    a. If yes, did you actually do it? How did you fund it?
    b. If no, why not?
5. When you are planning a clinical trial, how do calculate the budget?
    a. Did you get help with calculating the budget (e.g. from a Clinical Trial Unit)?
    b. Did you use a budget calculation tool?
        i. If yes: Which calculator did you use? How satisfied were you with the tool? What were the strengths and weaknesses of the tool? What could be done to improve the tool?
        ii. If no: Did you use your own template or did someone else (e.g. from a CTU) calculate the budget for you? What were the strengths and limitations of the approach you used?
    c. Did you consider empirical cost data from a similar clinical trial when calculating the budget?
        i. If yes, from where did you get the empirical cost data?
6. What is important to you when calculating the budget for a clinical trial? What would the ideal procedure and ideal budgeting tool include?
7. What were the main challenges to fund the trial?
    a. Where did the funds for your clinical trial come from? (proportion for each funding source)
    b. Did you have the clinical trial "fully funded" when recruiting the first patient into the clinical trial?
        i. Do you think the Swiss funders have a policy of fully or only partially funding clinical trials? Does this influence how much funding you request?
    c. Did you have to compromise on any aspects of your clinical trial because of funding limitations? What did you compromise on/sacrifice?
    d. What would you estimate was the proportion/total of "hidden costs"? That is, work you had to do that isn't reimbursed/funded e.g. feasibility work, resource use that was necessary to write the protocol and/or funding proposal, discussions with peers or reviews by experts, or establish a trial team/network?
8. How did you monitor costs during the trial? Did you use any tool?
    a. If yes, which tool did you use? How satisfied were you with the tool? What are the strengths and weaknesses of the tool? What could be done to improve the tool?
    b. If no, did someone else (e.g. from a CTU) monitor resource use and costs for you? Would you like to have a tool available? What functions should such a tool have?
9. From your experience, are the actual costs of an RCT often higher or lower than the estimated/planned costs or mostly on budget?
    a. If costs are higher, what were in your view the reasons for that?
10. In general, what do you think are the main cost drivers of a clinical trial? From your experience where could/should costs be saved?
11. In general, what would you suggest to facilitate/improve the acquisition of funds for clinical trials in Switzerland?

Funding agencies interview guide

1. To start, could you tell me a bit about the types and how many investigator-initiated clinical trials applications you receive each year?
    a. How many of these are single or multi-center clinical trials?

…

4. How important do you think feasibility work/pilot studies are before clinical trials are conducted? What do you think the role of Swiss funders are in supporting this work.
5. How accurate are clinical trial budgets from applicants? How often do you think planned costs match the actual clinical trial costs?
    a. Do you provide a budget calculator for applicants of clinical trials?
        i. If yes, which one? Why did you choose this one? What are the strengths and weaknesses of this calculator? What could be done to improve the tool?
        ii. If no, how do you expect applicants to develop their trial budget?

© 2021 McLennan S et al. *JAMA Network Open.*

b. Do you think that clinical trial budgets should consider empirical cost data from similar previous trials? How often does that currently happen?

c. Do you seek/obtain feedback from applicants regarding the actual costs of a trial after its completion?

6. How do you judge whether a clinical trial budget is appropriate or excessive? Do you use explicit criteria?

a. If yes, what are the criteria? Do you ask reviewers to comment on the appropriateness of the requested funding amount and leave the judgement to them?

b. Do you think that explicit criteria or suggestions of plausible ranges for requested funds for certain tasks would be desirable?

c. Do you think that more standardized terminology for budget categories/cost items for clinical trials in Switzerland would be desirable?

7. What is important to you as a trial funder regarding budget calculations of trial investigators? What would the ideal procedure and ideal budget calculation tool include?

8. How should costs be ideally monitored during a clinical trial? Who should do this monitoring?

9. Do you typically aim to fully fund an entire clinical trial or do you rather have a policy of providing partial support of clinical trials?

10. What would you estimate is the proportion/total of "hidden costs"? That is, work investigators do that isn't reimbursed/funded e.g. feasibility work, resource use that was necessary to write the protocol and/or funding proposal, discussions with peers or reviews by experts, or establish a trial team/network?

11. In general, what do you think are the main cost drivers of a clinical trial in Switzerland? From your experience where could/should costs be saved?

12. In general, what would you suggest to facilitate/improve the acquisition of funds for clinical trials in Switzerland?

Trial support organizations interview guide

1. To start, could you tell me a bit about the types and how many investigator-initiated clinical trials your organization supports each year?

a. How many of these are single or multi-center clinical trials?

…

4. How often do you consider conducting a pilot trial/feasibility work?

a. If often, how often does it actually happen? Who is/should fund pilot trials?

b. If rare, why not?

5. When you support a clinical trial with one or more services, do you typically calculate the budget for that trial too or is this a task the PIs do themselves?

a. When you calculate the budget for a clinical trial, which budget planning tool do you use? Did you develop the tool yourself, did you buy one, or is it a publicly available tool? How satisfied are you with the tool? What are the strengths and weaknesses of the tool? What could be done to improve the tool?

b. Do you consider empirical cost data from a similar trial when calculating the budget?

i. If yes, from where do you get the empirical cost data?

6. What is important to you as a trial support unit when calculating the budget of a trial? What would the ideal procedure look like?

7. From your experience, what are the main challenges to fund an investigator-initiated clinical trial?

a. From where do most funds for investigator-initiated trials in Switzerland come?

b. How often do you encounter the situation that a clinical trial is not "fully funded" when recruiting the first patient?

c. From your experience, how often do investigators have to compromise on any aspects of a trial because of funding limitations? What is typically compromised on/sacrificed?

d. What would you estimate was the proportion/total of "hidden costs"? That is, work investigators do that isn't reimbursed/funded e.g. feasibility work, resource use that was necessary to write the protocol and/or funding proposal, discussions with peers or reviews by experts, or establish a trial team/network?

8. Do you monitor costs during a clinical trial?

a. If yes, which tool do you use? How satisfied were you with the tool? What are the strengths and weaknesses of the tool?

b. If no, why not? Would you like to have a tool available? What functions should such a tool have?

9. How often do you encounter situations that planned and actual costs of a trial differed? In your view, what are the typical reasons for this difference?

10. In general, what do you think are the main cost drivers of a clinical trial? Where could/should costs be saved?

11. In general, what would you suggest to facilitate/improve the acquisition of funds for clinical trials in Switzerland?

Ethics committees interview guide

1. To start, could you tell me a bit about the types and how many investigator-initiated clinical trials applications you receive each year?
    a. How many of these are single or multi-center clinical trials?

…

4. To what extent do ethics committees look at clinical trials budgets/funding?
    a. Do you think the underfunding of clinical trials is an issue?
    b. What role do ethics committees have in preventing underfunded RCTs? Should ethics committees do more regarding this issue?

5. We have evidence from several empirical studies that estimates about patient recruitment and trial duration are often overoptimistic and pilot studies (including internal pilots) are very rare. How important do you think feasibility work/pilot studies are before clinical trials are conducted?
    a. If important: What should be done to change this? Which stakeholders would be best suited to promote and endorse feasibility work/pilot studies?
    b. If not important: How could trial investigators then come up with more realistic estimates about patient recruitment and trial duration?

© 2021 McLennan S et al. *JAMA Network Open.*

Supplemental tables

eTable 1. The Problem

Theme		
**Code**	**Subcode**	**Example Quote**
Underfunded IICTs		*"The problem is all the clinical trials are dramatically underfunded. And that is a real problem." P29 Clinical Trial Funder*
Compromises made because of funding limitations	Reducing sample size	*"Some investigators have to reduce their number of patients and then the power of their trial to be able to stop their trials." P19 Clinical Trial Support*
	Adapting end point	*"Well, they sacrifice the end point. Yes. Because, so we do/ They have their end point, they have their question, we do a first power calculation. There is way too many people, so then we start adapting the end point to get down to a feasible… Well, or we say: "Well, let us do a pilot."" P10 Clinical Trial Support*
	Length of follow-ups	*"Yes, sure. Probably the length of follow-ups is one of the main issues. We would wish for a longer follow up." P4 Primary Investigator*
	Project management	*"Project management is a whole. You know, not only site management but managing the whole project. Monitoring the progress, monitoring the budget. Ensuring communication of all stakeholders within the project. All these kinds of things that the project management does." P12 Clinical Trial Support*<br><br>*"Yes, actually honestly, the first thing we cut is monitoring." P24 Primary Investigator*
Hidden costs	Significant amount of trials costs not reimbursed	*"Well, you know, it's difficult to estimate but probably it's around 15 to 20 percent." P5 Primary Investigator*<br><br>*"So, I would say it's about 10 to 20 percent maybe of the costs of the grant." P32 Primary Investigator*<br><br>*"I would say it would be a 20 percent job for about one year." P39 Primary Investigator*<br><br>*"Yeah, yeah. The hidden costs are always much higher than budgeted […] So, I would estimate the amount of hidden costs somewhere around maybe 20 percent over budget, but that's because we already are including funding for pilot studies and for quality management, data management, etcetera. People who don't sufficiently include that, then costs would be up by 40 percent of the budget. I think, all trials basically are always underfunded. There's a huge underfunding problem in clinical trials. I mean, especially investigator initiated." P46 Clinical Trial Support*<br><br>*"It's a problem, of course. Or one of my real problems is…me, personally, I am employed 100 percent here at the [hospital]. And so, my own work never is funded somehow, or I can't really understand how it could be. So, I only can get funding for my students, for my machineries and postdocs, but not for myself. So, whatever I do, I do for free. That's somehow a little disturbing. But*

© 2021 McLennan S et al. *JAMA Network Open.*

		I'm paid here anyway. I guess the [hospital]' pays something to these trials, even though they don't know about this." P39 Primary Investigator
	Unfunded work often not recorded	*"I think a lot there. A lot. A lot. They are not aware of how much they actually put, because they are never recorded, that is the problem. That is also the difficulty we are facing with, it is another discussion, but we record everything more or less. So, we have a quite good picture of what time do we need to invest and what time we need to provide the service, whereas the investigators, they do not. They are not aware of how much they actually worked. Or not explicitly. They implicitly know, but they do not realize. And then, also they have the others. Their co-workers and everybody." P12 Clinical Trial Support*

© 2021 McLennan S et al. *JAMA Network Open.*

eTable 2. The Reasons

Theme		
**Code**	**Subcode**	**Example Quote**
**Inaccurate budget estimates**		
Poor planning and preparation	Lack of professional support with cost management	*"This was horrible, actually. […] I had no idea about budget. And I, of course, asked the head of my department and then our clinic manager. But what I realized afterwards was that I also forgot to budget some other stuff. And I was really very modest. […] Afterwards, you always know better. A colleague from Berne did the budget together with his [CTU] which was more appropriate than I did because I kept it just in my own department. They were, of course, more experienced. And they took care of some points, that I really didn't consider in my grant." P18 Primary Investigator*<br><br>*"No, I don't really monitor. I mean, the grant money is being managed by the university. We've an account. At the end of the year, they send a financial report today to the National Science Foundation. But essentially, all I do is once a year roughly, I try to understand whether or not there is money left. But I have absolutely no overview of what's going in, what's going out." P39 Primary Investigator*<br><br>*"Our experience is that if the investigator is doing this by himself that it is never matching. This is far, far too low." P26 Clinical Trial Sponsor*
	Unsystematic and limited evidence-base	*"It really depends. In some cases, I think [feasibility work is] a waste of time, you should rather go directly to the real." P35 Primary Investigator*<br><br>*"Actually, not others […] But we are looking basically in our own data. But not formalized." P12 Clinical Trial Support*<br><br>*"No. Just the experience of two colleagues." P18 Primary Investigator*<br><br>*"Well, actually, if they [empirical cost data] are available, yes. But so far, I had difficulties to finding such cost data. I'm actually not even aware where I could find them…" P32 Primary Investigator*<br><br>*"I don't think that [empirical cost data] would be very helpful if this has not been done in Switzerland. Because health care systems are so different, funding agencies and you know, and then the cost." P8 Primary Investigator*<br><br>*"No. At least I never had a situation where I felt that it [empirical cost data] was so comparable to our setting and what we do that it's useful." P4 Primary Investigator*
Unforeseeable events		*"…we had once during the study the company who is making the drug telling us "Maybe the delivery will be delayed by three months." I said: "What? Will be delayed by three months?! I cannot stop my study for three months, you know"." P18 Primary Investigator*

© 2021 McLennan S et al. *JAMA Network Open.*

		"This is a good question, we're only actually really seeing now a bit the discrepancies […] And we definitely see that most of them communicate us that they were short on money. Some have solved the problem by themselves. They just applied for some other funding or like some side projects and cross-funded it. But I think my general impression is that for such long projects, there's just so many unknowns, that everyone is running out of money at one point. But I think, that's also somehow a question we might have to address in the future. Because, if you do that in industry, your budget / Like you're 10, 20 percent on top, and we do not allow that. We say, you know, "you justify every single cent". "You justify why you need that". And the justification is not for unforeseen costs." P48 Clinical Trial Funder
Intentional underestimating budgets		*"Nearly always it's an underestimate of the costs, because you have a kind of feeling, what is the upper limit of money you'd get from this funding agencies? And you make the budget, so that it fits in this imaginary upper limit." P40 Clinical Trial Funder*<br><br>*The strength is clearly that we are often matching. And we have no conflict of interest. I mean, we calculate what we need. An investigator will calculate what he thinks he may get as a financial support. So, if his experience is that he usually gets 350.000 from the grant provider, he will squeeze his entire trial into 350.000." P26 Clinical Trial Funder*<br><br>*"I think, it is simple. When I take the budget I try to sell myself as cost-effective as possible." P2 Primary Investigator*
Limited assessment and oversight of budget	Very superficial assessment	*"Most of the discussion, like 90 percent, would be on scientific content, the budget really has a very little role in the discussion." P40 Clinical Trial Funder*<br><br>*"It's more scientifically than the budget, which determines how much money is given IICTs […] So we didn't look, at least we as scientists - maybe the administrator looked in more detail – we looked quite superficially at the budget." P35 Clinical Trial Funder*<br><br>*"…it's not our crucial issue really to judge on the budget. So we really don't focus on it." P3 Ethics Committee*<br><br>*"…for the ethics committee this is not possible to do. We just accept what they are submitting" P14 Ethics Committee*
	Lack of explicit criteria	*"No, but we, based on the experience of the committee and they are all quite experienced people. I think we can get a good guess." P11 Clinical Trial Funder*<br><br>*"So, we rely on the judgment of our panel and that is in some ways problematic, because our panel is international and they also have a hard time judging sometimes if the budget is appropriate for Switzerland." P48 Clinical Trial Funder*
	Budget accuracy responsibility of the PI	*"Well, we want to see that this is feasible but we also leave it in part to the responsibility of the investigators." P11 Clinical Trial Funder*

© 2021 McLennan S et al. *JAMA Network Open.*

		"We will mention that and say "You have to look for that.". But we will not reject the project because of this, because these things are in the responsibility of the researcher and not of the ethics committee." P3 Ethics Committee
**Funding limitations**		
Limited funding sources	SNSF´s IICT program main option for full funding	*"But I think that really was a game changer, that you could come and you could get actually full funding for a clinical trial or at least for certain ones." P48 Clinical Trial Funder*
	Foundations only able to fund pilot studies or provide partial support	*"I think most of the private foundations they do not have enough resources to fund a clinical trial by their own." P2 Primary Investigator*<br><br>*"Well, as I said, a full budget for a large clinical trial is beyond scope of [Foundation], it is not possible. But, of course, you can do a pilot phase. You can start up with just maybe a few dozens of patients for within that range of one center maybe, for 100.000 Swiss francs. Yeah, that's fine." P31 Clinical Trial Funder*<br><br>*"It should be also for promotional reason and marketing reason, it should be a [Foundation] supported project. So, they never would fund, let's say, you have a pharmaceutical project and you would like to add something. The [Foundation] would say no […] Or we have other things which we reject and I give you an example. A larger multi-centre trial, for example in Germany, you know there was in big Germany, and they would like to add Swiss Centres to their multi-centre investigator-driven trial, and the authors ask for support from the [Foundation] for the Swiss centres. We generally said no because at the end it will be 85 percent of the authors will be from Germany, 10 percent end up to be Swiss, maybe two people. And at the end it is a project of the "Deutsche Forschungsgemeinschaft" and the Swiss [Foundation] is at the end somewhere. Such things have been rejected even if the project was good because the [Foundation] does not see an own identification, the marketing issue. […] Because we thought there is no autonomous appearance at the end for Switzerland and the [Foundation]." P29 Clinical Trial Funder*
	IICTs often started without having secured sufficient funds	*"I mean, we have some plans, some budget plan, but we knew that it was not enough. So, we had to get additional funding. And you know, the experience is that once you started the trial you have your first patients in, it is easier to get someone to jump on the trial because they know you have already done a lot of the basic work, and the trial is going to be done. And so I think you still have good possibilities, once you start a trial to get more funding." P7 Primary Investigator*
Unrealistic expectation of funders	Gross underestimation of costs	*"I don't know if it's a real policy but I think it's a gross underestimation of the cost of trials. I think that's probably more the issue. I don't think that they on purpose only fund it partly, but they grossly underestimate the resources needed I'd say." P4 Primary Investigator*

© 2021 McLennan S et al. *JAMA Network Open.*

		"The policy is to ignore, that much more would be needed. I think it's ridiculous." P38 Primary Investigator<br><br>*"I don't know. I think it's not their intention that they don't fund the full trial, but it's the problem that they don't understand, that monitoring for instance or other quality measures/ I mean, it's not for free. And it's a service that has to be paid, like a lab service or material that you ordered. But it's nothing you could hold in your hands and I think it's this problem, why funders don't pay for things like for quality. Because they're not aware of this." P6 Clinical Trial Support*<br><br>*"It's not easy. In most funding agencies, if you make a realistic budget, the council there will tell you, that you're far too expensive, that you're doing luxurious research, that you ask much more than other researchers for the same thing and that your project is less value for money. Particularly in the less professional funding agencies, many people in the council are not professional researchers, so they think that much of the work can be done just during evenings and weekends. And they don't have a realistic experience of how much money is necessarily needed to pay people salaries for what they are doing. I always under-report the costs, because personally I think, otherwise they think I'm too expensive. So, I try to keep sticking to the average, even if I know, it will cost me more time." P40* Primary Investigator
	Funders cutting budgets	*"Several years ago I asked for a budget, it was cut by 50 percent by the SNF. So to do the same trial with 50 percent less budget, which was impossible. So I had to get a lot of additional funding to conduct this trial." P20 Primary Investigator*<br><br>*"Well, yes, of course, they cut my budget. And then people told me "They always cut." And I was like, "Oh shit. If I knew that before I would have quite put more, that if they then cut something, I would still be OK […] [Actual costs are always] Higher, I mean it starts already with the cutting from the [Foundation]. We did not put anything on the budget that we don't need. They cut a part of the deal, which means that we had to compensate for it somehow." P18 Primary Investigator*<br><br>*"I'm honest with my budgeting. And I don't inflate my budget just because, you know…It looks like when the [Foundation] tells you, "Yeah, do the same with 30 percent less.", they give you a signal that you should play with your budget. You're over-inflated. And I think it's a very bad message because for the next time you inflate your budget. Why do that shit? Come on. 30 percent more. I mean, they were able to do this computation. And I don't get it. I don't get it. You want to be honest. And if people don't spend, then they give it back. And that's how it should be. I think that's the major concern I have. I don't get this way of working. And I experience that each time they cut the budget. For no reason. You're overestimating. […] We can really say, OK, the budget gets peer reviewed by people who can use data and say: This is not OK. You have put 10 times more cost on*

		this MRI than is usually done, or "You say, you do a questionnaire, but you use 300 percent more study nurses than ours did. There's something off in your calculation, please revise it. But not just say, "You know, you're playing with the rules. So, we'll just cut it by 30 percent.". I think it's unfair." P13 Primary Investigator<br><br>*"Yeah, we hear that quite often and in some way we are puzzled, because this is exactly what we want the funding program to be there for, to actually fund trials with the costs they have. And I mean, there was this initial budget of ten million given randomly in some way. And unfortunately, in the last five years, we never had a reason to say, "we need more money". And they're always calling and asking, "but I'm thinking, maybe four million is needed. Can I do this"? Yes, please apply with what it costs. Because ultimately, if we have no arguments to ask for more money from the government, we will not ask for more money. And I think that is something that we have to work with them together […] But as much as we say, "apply with what it costs", I mean, we still haven't seen more than like four point something million. And that if from the political side they ask us, "how much is the trial costing", well, we can only see what they have applied for. And that seems to be not the truth, but we cannot change it […] And I think, we may have caused in the beginning, because we said, we have ten million and we wanted to fund at least four trials. And I think, that often led to the result that people apply with 2.5 million. And now luckily, this has changed, they applied for higher budgets, but they still seem to have in their mind, that there is some kind of a cap." P48 Clinical Trial Funder*

© 2021 McLennan S et al. *JAMA Network Open.*

eTable 3. The Solutions

Theme		
**Code**	**Subcode**	**Example Quote**
**Improving planning and quality**		
Support of clinical researchers	Ensuring clinical researchers have sufficient time for their IICTs	*"If you get money for such a trial, that you as the PI could just to reduce your clinical duties to some degree, even if it's just 10 or just five percent. It would be really helpful. Otherwise we often have these time constraints that you actually should do some clinical work, but you should actually work for the trial. This is a conflict of interest…" P36* Primary Investigator<br><br>*"If you do a clinical training it is hard to do academia […] if you do research, nothing, there is no support. You do everything in your spare time. And I think that is not good. Because if we lose our academic registrars to do it, I mean because they do not have the time, it is not good. We need to find ways to support young clinical researchers. That is one of the things, I think, we should really put some effort into it. And think of ways how they can do their clinical training together with a good academic career." P2 Primary Investigator*
	Education and training	*"I think a little more education on the medical community on how they need to be done. Though there is a couple of clinical trials is not everywhere present. There is not enough education on it, on the process, on the regulations, needs little more efforts." P22 Primary Investigator*<br><br>*"Education, training and there must be a development path for academic, clinical researchers in the big hospitals." P34 Primary Investigator*<br><br>*"I think that the problem we have here in Switzerland is, that we have a very traditional and strong basic research. But often our junior people don't know how to write a trial protocol or a protocol for a clinical research project. I think we should offer classes and courses how to improve our grant writing ability. I think that would also increase our probability to get funding then." P32 Primary Investigator*
Clinical trial support	Improving clinical trial support organizations	*"No, maybe I really would like to point out: harmonization at least within the big CTUs in Switzerland makes a lot of sense and there should be exchange and maybe we could kind of put forces together and so try to improve the situation." P6 Clinical Trial Support*

© 2021 McLennan S et al. *JAMA Network Open.*

		"Absolutely. I think it would also be really helpful in the frame of the Swiss clinical trial organization. This will be a point to be discussed at the next retreat of the board of CTU directors. […] The plan was, to do an exercise, with the same clinical trial and the same study and so on. And look: What comes out in the different CTUs, regarding the planned costs. And there is already clarity, that there are probably different systematics. So, it would definitely be helpful to harmonize the approaches." P41 Clinical Trial Support<br><br>*"We need probably more funding for CTUs. So, the question of CTUs, how they are funded. So probably, parts of the CTU, a big part of the CTU need to be funded by the university, and the hospital. And probably, some kind of just grants from the university or the hospital for making the collaboration with the CTU less expensive or even free." P21 Primary Investigator*<br><br>*"You know, my favorite model would be that the funding agencies pay kind of a – how to say that in English – like a "Sockelbetrag", you know, kind of a standing budget to the local CTUs and in turn the local CTUs review all the projects that apply for the grant. That would be great, because then you know we would have the resources to do all of these reviews, to give input. To help them improve. And, you know, this work is paid and kind of acknowledged also, yes." P6 Clinical Trial Support*
	Developing support tools	*"No, as far as I know, no. And I think it would be very helpful. Yeah, that could be a great tool, you know, for researchers. Just to think about it, because also, if you're not familiar you forget […] and this can be very expensive, right." P17 Clinical Trial Funder*<br><br>*"If there is a good budget calculation tool, that would be helpful. Typically, I just go by the past clinical studies that I did and try to find out think about where I forgot something and what needs to be added." P35 Primary Investigator*
Networking and guidance	Sharing knowledge and experiences	*"And then, what I really would appreciate I mean, we were among the first to get such an IICT - is like an exchange between the PIs. Because here in [city] there are two other IICTs […] and we all made the same experiences. And we talked to each other to support us. But if there would be really an exchange of information, and I have to say when I tried to give the feedback to the SNF I do not have the feeling that anyone is interested or that they want to organize a meeting where all the PIs*

		come together that share the experience with the IICTs." P16 Primary Investigator<br><br>*"Well, too often, to be honest, and I really think and this is an important point; I really think that there are not enough platforms where investigators can go to and actually really get help. […] There's still too many cooks out there doing their own thing. I'm a strong defender on getting people together and trying to build that synergistic use of platforms, of tools, exchange of knowledge and, you know, have teams which support trialists." P46 Primary investigator*<br><br>*"One, definitely, I find, this is the networking that we're lacking and also kind of a more centralized…I think with the SCTO now, we have many good initiatives and people get to know each other better. But there is a lot of knowledge in Switzerland that is just currently not being shared." P48 Clinical Trial Funder*
	Sharing cost-data	*"Yeah, it's a very good point. I mean, we have the problem of the data privacy. So, we were unable really to share anything in this regard. But we were also thinking to create maybe a bit more general input. But then again, general is not helpful in most cases because they really want to know how much I need for that. So, as we cannot really share this data, we were planning to have maybe a bit more help to set up workshops from our side, where then the trialist from the trials, that are right now funded through us, can come and speak to people that are interested […] So, it's a bit the question, like what is our role? But we've decided for us, I mean, no matter what really our role is, our interest is to get good proposals." P48 Funder*
	Information sources	*"It would be nice to have kind of a site, an internet site, where you see these are funders of clinical trials, you know. […] That would be something very helpful. And maybe resources, you know, people that you can talk to, that have experience I guess. Most time have CTU that they can connect to. But I think, just that you would see who would actually fund clinical trials, you know." P8 Primary investigator*<br><br>*"Well, actually, the problem of our system is that there is the Swiss National Science Foundation. And then there are lots of private foundations that are national, but many are local foundations. So, if you would have sort of a source where we can see what type of foundation or other funding source is offering money for which projects, I think that would be very helpful to have some sort of general*

© 2021 McLennan S et al. *JAMA Network Open.*

		oversight about the funding situation in Switzerland." P32 Primary investigator<br><br>*"I think so. I mean, for very inexperienced researchers who have no idea how much a research scan or an MRI scan costs, I think it would help to provide that sort of range. How much things cost." P31 Clinical Trial Funder*<br><br>*"So, what we definitely never wanted to give was a range of how much a trial is allowed to cost, because we think that's just not appropriate. But how much different parts might cost is a good idea." P48 Clinical Trial Funder*
**Cost savings**		
Not possible to save costs		*"In my clinical trials? No. There is no cheaper version of the trial. I think if the pharmaceutical industry would do the same, I think or I came to the conclusion that they would spend like at least 10 times more for the same thing. I cannot imagine how costs could be saved." P39 Primary investigator*<br><br>*"The problem is even in 2020, as I said, the trial budgets are even not at a level where I would say we have sufficient funding for good quality conduct. So, the trials that we are doing are far too cheap. So, I do not think we can talk about it, but that is my opinion. Sure, it is all my opinion. But there is no room for saving money, because they are very cheap the trials that we are doing." P12 Clinical Trial Support*
Possible cost savings	Harmonising and simplifying bureaucracy	*"No, that's certainly a good point. I mean, it's too much bureaucracy and administration, in clinical trials, and there, you could maybe save money." P28 Primary investigator*<br><br>*"I think we should reduce the regulation and the costs, basically, because the problem is: You have now the same regulations for clinical studies. If it's a drug company, or if it is just that one investigator-initiated study, that wants to do a small study about whatever. Then you have the same regulations, and this is just not payable. My view is that we should reduce the costs of the regulation, which is probably not happening." P30 Clinical Trial Funder*<br><br>*"The other thing is the ethics approval process. I mean, it is already quite good to have the lead agency, but I still think it is ridiculous that it has to be re-submitted to each Canton. I mean, if you have a Swiss National Science Approval, why do you need to have different plain language statements for every Kanton? This is just ridiculous. I mean, this is, yes, I mean, you*

© 2021 McLennan S et al. *JAMA Network Open.*

		lose a lot of hours in doing so. So, that the Swiss National Science Foundation negotiates with every ethics committee in Switzerland, says: "Well, these projects they are approved by the Swissmedic, and there is one plain language statement for Switzerland." I mean, this is ridiculous, but that is my point of view. Zurich is not different to Basel, and not different to Geneva, you know what I mean. These are the things where I think money can be saved." P2 Primary investigator
	Simplified and remote techniques for quality monitoring	*"You could probably save costs for finding more intelligent ways to do quality management to use remote tools to control for certain issues. […] Wherever you can use remote techniques to control for quality, I think you could save a lot of costs. I already told you that the staff is the most important cost driver. But from a CTU side it is monitoring. It is people traveling to the centers and checking whether everything is done the right way. This is very important, very expensive, but I think it is just does not make the trial significantly better. It is the way it is done right now. So, you could save some money there." P10 Clinical Trial Support*<br><br>*"You could save money, especially in leaving out, or significantly simplify monitoring. Just that, because I don't think it really makes a difference. So that's another comment, because usually it's only confusion and doesn't really improve data quality." P28 Primary investigator*
**Increasing funding**		
Making sufficient funding available	Increasing public funding of IICTs	*"Well, I think the first one would be to increase appropriate funding by the Swiss National Science Foundation. That is inadequate and the funding available for clinical trials is in a range that does not enable large scale "clinical trials". The budget that are provided by the SNF simply don't suffice." P5 Primary investigator*
	More flexibility with use of funding	*"Yes, yes. Two more things she said: More topic specific calls, and less formal criteria or more flexibility for criteria like the length of a trial, the activation date of a trial, that is limiting a lot." P26 Clinical Trial Funder*<br><br>*"They should have more flexibility. What we always think is that five years for a useful clinical trial is too short. Something like Covid, some of your key player in your team broke his leg. You always have something which is unforeseen. There are so many unpredictable aspects. For longer trials, why not have a lot of trials have more flexibility? This is the budget. If it takes one year longer you maybe have some extension of a year. It would be really important, that clinical*

		trials could be finished to avoid this research waste." P36 Primary investigator

© 2021 McLennan S et al. *JAMA Network Open.*
